# A Background-Free SERS Strategy for Sensitive Detection of Hydrogen Peroxide

**DOI:** 10.3390/molecules27227918

**Published:** 2022-11-16

**Authors:** Kaixin Chen, Haoling Chen, Songxian Liang, Jindan Wu, Ping Zhou, Nan Li

**Affiliations:** Key Laboratory of Biomaterials of Guangdong Higher Education Institutes, Department of Biomedical Engineering, Jinan University, Guangzhou 510632, China

**Keywords:** Au microparticles, azo reaction, background-free SERS sensor, H_2_O_2_-responsive Raman reporter, ultrasensitive H_2_O_2_ detection

## Abstract

The accurate and sensitive detection of biomolecules by surface-enhanced Raman spectroscopy (SERS) is possible, but remains challenging due to the interference from biomolecules in complex samples. Herein, a new SERS sensor is developed for background-free detection of hydrogen peroxide (H_2_O_2_) with an ultralow detection limit (1 × 10^−10^ mol/L), using a Raman-silent strategy. The Au microparticles (Au-RSMPs) resembling rose-stones are devised as SERS substrates with a high enhancement effect, and 4-mercaptophenylboronic acid (4-MPBA) is selected as an H_2_O_2_-responsive Raman reporter. Upon the reaction with H_2_O_2_, the phenylboronic group of 4-MPBA was converted to a phenol group, which subsequently reacted with 4-diazonium-phenylalkyne (4-DP), an alkyne-carrying molecule via the azo reaction. The formed product exhibits an intense and sharp SERS signal in the Raman-silent region, avoiding interference of impurities and biomolecules. As a proof-of-concept demonstration, we show that this SERS sensor possesses significant merits towards the determination of H_2_O_2_ in terms of broad linear range, low limit of detection, and high selectivity, showing promise for the quantitative analysis of H_2_O_2_ in complicated biological samples.

## 1. Introduction

Surface-enhanced Raman scattering (SERS) spectroscopy is a highly sensitive technique that enhances the Raman scattering of molecules supported by some nanostructured materials [1]. SERS allows for high specificity because it reveals the vibrational fingerprints of the analytes [2]. Compared with other detection techniques for biomolecules, SERS simultaneously possesses several key merits for bioimaging and biosensing, including its ability to characterize vibrational peaks, insusceptibility to photobleaching/photodegradation, negligible interference of water, multiple detection capabilities, and unmatched detection sensitivity [3,4,5].

Quantitative and precise measurements of metabolites or biomarkers in biological samples are critical in fundamental research and clinical diagnosis [6,7,8,9,10,11,12,13]. Among them, some SERS-based methods have been developed; however, it remains a challenge to differentiate traditional Raman tags or reporters from biomolecules since the proteins and lipids can generate a strong background signal in the “fingerprint region” (<1800 cm^−1^). To accurately assess metabolites in complex samples, a new concept of “silent-region” Raman reporters was reported [14,15,16], which shows the characteristic Raman peaks in the range of 1800–2800 cm^−1^; most of the biomolecules do not generate any Raman vibrational signal in this region [3].

To date, “silent-region” Raman tags and reporters were mostly applied to SERS imaging focusing on tumor cell targeting [17] and therapy [18], tissue, and living-cell imaging [19]. There are several reports based on SERS “silent-region” measurement for physiological detection [20,21,22,23]. However, these SERS sensors suffer from two major drawbacks: (1) the nanoscale SERS substrates lack high enhancement for trace detection with poor reproducibility and signal instability [24,25,26,27]; (2) the extensive application of “silent-region” reporters is impeded due to the complicated chemical synthesis or bioconjugation [3,28].

We herein demonstrate a new probe for H_2_O_2_ detection through the development of a highly-enhanced SERS substrate and a “silent-region” Raman reporter. The SERS probe contains “rose-stone-like” Au microparticles (RSMPs) and an H_2_O_2_-responsive Raman reporter. The as-synthesized RSMPs can generate intense SERS enhancement for the ultrasensitive determination, while the Raman reporter can produce the silent-range Raman signals upon the reaction with H_2_O_2_, enabling the detection of H_2_O_2_ with high sensitivity and selectivity.

## 2. Materials and Methods

### 2.1. Reagents and Materials

HAuCl_4_·3H_2_O, HCl, Polyvinylpyrrolidone (PVP, Mw = 40,000 Da), 4-mercaptobenzoic acid (4-MBA), 4-mercaptophenylboronic acid (4-MPBA), hydrogen peroxide (H_2_O_2_, 30%), NaNO_2_, 4-ethynylaniline (4-ENA), NaBF_4_, *N*-methyl pyrrolidone (NMP), and ethanol were purchased from Sigma. *N*-(3-Amidino)-aniline (NAAN) was synthesized according to the reported method [29]. All reagents were analytical grade and used as received. Deionized water (DI) was acquired by a Milli-Q water system (18.25 MΩ·cm) and used for all the aqueous samples.

### 2.2. Characterization Conditions

Field emission scanning electron microscopy (FESEM, Ultra-55, ZEISS, Oberkochen, Germany) was used to study the morphology of RSMPs. The size of RSMPs and their standard deviation (SD) were calculated by measuring over 100 individual particles. Laser Confocal Micro Raman Spectrometer (LabRAM HR Evolution, HORIBA Jobin Yvon S.A.S, Bensheim, Germany) was employed to measure the signal of SERS spectra by using a 633 nm laser excitation source. To measure the SERS signals, 1.5 μL SERS particles were dropped on a glass slide, and the measurement was conducted on a single particle after being dried in the air.

### 2.3. Synthesis of RSMPs

The RSMPs were prepared via the reduction of HAuCl_4_ using NAAN as a reducing agent [24]. Simply put, 25 μL 16 mg/mL PVP (0.40 mg/mL) was added into 1 mL 1 mM HCl solution, followed by the addition of 8 μL 10% HAuCl_4_ solution (2 mM, dissolved in 1 mM HCl). After cooling the mixture to 4 °C, 50 μL of 100 mg/mL NAAN (16 mM) in 1 mM HCl was injected, the mixture was stirred for 30 s and allowed to react at 4 °C for 24 h without further stirring. The final solution was centrifuged at 6000 rpm for 5 min, and the collected pellets were subsequently washed three times with NMP and DI to remove the impurities. The eventual pellets were collected as RSMPs and stored in the fridge for further usage. To acquire the appropriate particles as a good SERS substrate, the concentrations of NAAN (3, 4, and 5 mg/mL), PVP (0.40, 3.20, and 12.80 mg/mL), and the reaction time (24, 72, and 120 h) were optimized.

### 2.4. Synthesis of 4-Diazonium-Phenylaklyne (4-DP)

4-DP was prepared according to the typical diazonium reaction. 4-Ethynylaniline (4-ENA) (100 mg, 0.59 mmol) was dissolved in 1 mL 1 M HCl solution and cooled by ice water. In another flask, NaNO_2_ (45 mg, 0.65 mmol) was dissolved in cooled DI water (0.25 mL), which was subsequently added to the 4-ENA solution dropwise. The mixture was left to stir at 4 °C for 1 h and room temperature for 30 min, followed by the addition of 2 mL NaBF_4_ saturated solution. The resulting product was filtered and the precipitate was collected and thoroughly washed with cooled water. After drying under a high vacuum, the 4-DP was stored in the fridge.

### 2.5. Preparation of SERS Probe

200 μL of RSMPs aqueous solution was mixed with 1 mL 1 mM 4-MPBA solution (dissolved in ethanol), and the mixture was stirred for 2 h by a vortex at room temperature. After washing with ethanol and DI, the 4-MPBA modified RSMPs were distributed in 50 μL DI water as the SERS probe. To measure the SERS performance of the resulting SERS probe, 10 μL of SERS probe solution was dropped on a cleaned glass slide and dried in the air, and the SERS signals on each particle were measured.

### 2.6. H_2_O_2_ Detection

100 μL H_2_O_2_ solutions with varying concentrations were incubated with the SERS probes for 1 h. After washing with DI water, the resulting SERS probes were dissolved in 20 μL DI, followed by the addition of 10 μL 100 mM 4-DP and stirring for 1 h. After washing with NMP and DI water, 10 μL of the eventual SERS probes was dropped on a glass slide for SERS measurements after being dried in air.

### 2.7. Interference Experiment

10 mM NaClO was prepared by mixing 10 mM NaOH and HClO solutions. 300 μL 10 mM Cu(NO_3_)_2_, FeCl_3_, and NaClO were mixed with 20 μL SERS probe solution. After 1 h incubation and washed with DI water, the SERS probes were mixed with 10 μL 100 mM 4-DP for 1 h. The SERS probes were washed successively with NMP and DI water. 5 μL of each SERS probe solution was dropped on a glass slide for SERS measurements after being dried in air. Each spectrum was processed by subtracting the baseline and normalized using the intensity at 1071 cm^−1^ due to the C-H stretching vibration of the benzene.

### 2.8. Real Sample Test

The standard additional method was applied to test the applicability of the resulting SERS probes. Human serum solution was centrifuged and the collected supernatant was diluted 1000-fold with PBS, followed by spiking with different concentrations of H_2_O_2_. After incubation of SERS probes with H_2_O_2_ solutions, and subsequently reacting with 4-DP, the SERS probes were collected and their SERS signals were measured.

## 3. Results and Discussion

**Figure 1:**
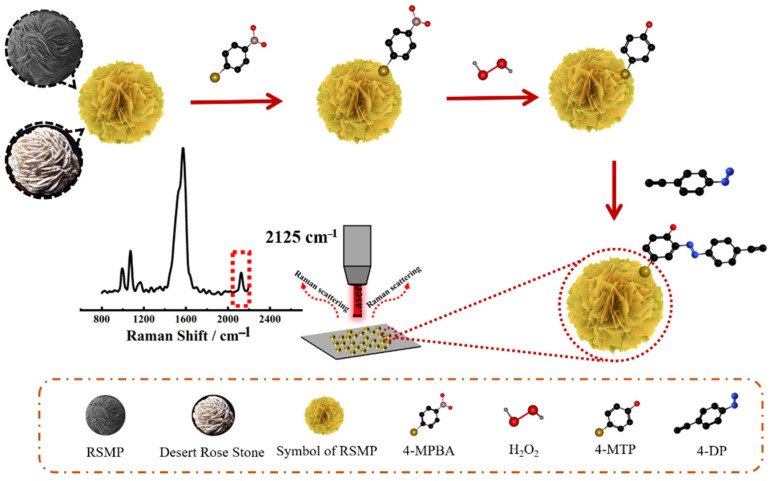
Schematic illustration of the procedure for H_2_O_2_ detection by SERS responsive probe with “Raman-silent region” reporter.

**Figure 2:**
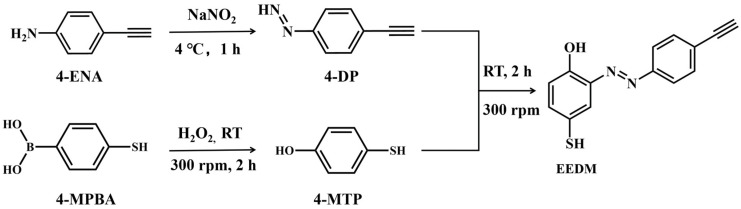
The synthesis of 4-DP and the mechanism of H_2_O_2_ detection.

The SERS sensing mechanism is shown in Figure 1. Firstly, we synthesized Au-based ”rose stone-like” microparticles named RSMPs due to their appearance which is similar to rose stones found in deserts. Their layered structure carrying multiple nanoplates provides abundant nanogaps and edges within RSMPs, which can generate hotspots for the SERS enhancement, resulting in the RSMPs being an excellent SERS substrate. 4-MPBA can be modified on RSMPs via the Au-S bond, and the reaction of 4-MPBA with H_2_O_2_ can transform the phenylboronic acid group into a phenol group [30]. Subsequently, 4-DP (4-Diazonium-phenylalkyne) can react with phenol to form an azobenzene compound carrying an alkyne group, which has a “silent region” Raman fingerprint (2125 cm^−1^). The amount of alkyne group on the SERS particle surface is dependent on the formation of the phenol group, which is determined by the H_2_O_2_ concentration, therefore, H_2_O_2_ can be profiled with significant sensitivity owing to the high signal/noise SERS measurement. Figure 2 demonstrates the synthesis of 4-DP and the H_2_O_2_-induced azo reaction. 4-ENA reacts with NaNO_2_ in acidic conditions to generate 4-DP. Eventually, the diazonium group of 4-DP reacts with the phenol generated by the reaction of 4-MPBA and H_2_O_2_ to form an azobenzene derivative EEDM ((*E*)-2-((4-ethynylphenyl)diazenyl)-4-mercaptophenol).

**Figure 3:**
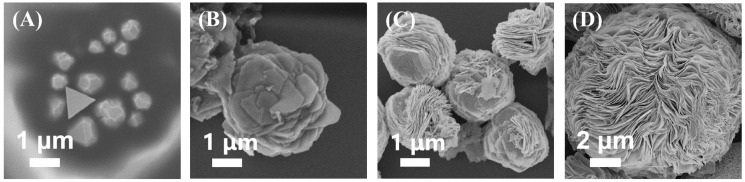
The time-dependent growth of RSMPs. (**A**) 10 min, (**B**) 1.5 h, (**C**) 4 h, (**D**) 24 h.

To study the mechanism of RSMP growth, samples at different time intervals were collected. In the early stage, HAuCl_4_ was reduced to form Au nanoplates by NAAN, while NAAN was oxidized to form PNAAN, which has a highly selective binding ability on the Au (111) facet, and the growth on this facet was inhibited, resulting in the growth of Au nanoplates. A mixture of cubic Au nanoplates with a diameter of 200–800 nm was observed after 10 min reaction (Figure 3A). As the growth continued, the Au nanoplates further grew into microplates (>1 μm) and new Au microplates were generated on the surface of prior plates to form ”rose stone-like” microparticles (RSMPs) (Figure 3B). After a 4 h reaction, RSMPs with a diameter of 2–3 μm were observed (Figure 3C), which further grew into the microparticles with an average size of up to 10 μm and exhibit densely-packed layer structures (Figure 3D).

**Figure 4:**
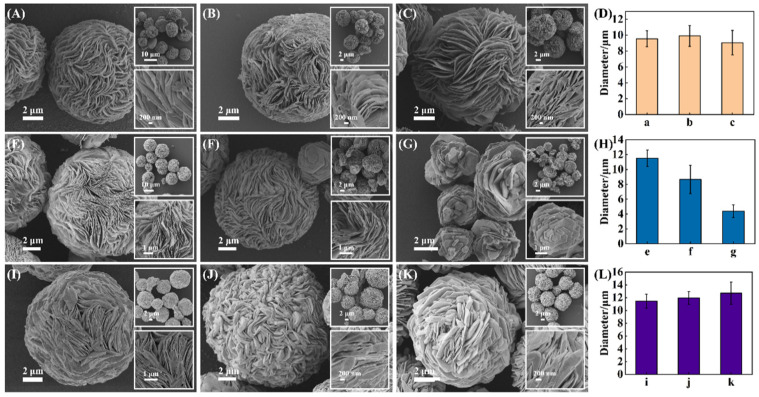
SEM images of RSMP prepared under different conditions. (**A**–**C**) RSMP prepared with 3/4/5 mg/mL NAAN, 1.6 mg/mL PVP, 24 h; (**E**–**G**) RSMP prepared with 0.4/3.2/12.8 mg/mL PVP, 5 mg/mL NAAN, 24 h; (**I**–**K**) The time of RSMP’s preparation was 24/72/120 h, respectively, with 0.4 mg/mL PVP, 5 mg/mL NAAN; (**D**,**H**,**L**) Histogram of the average diameter of the particles under different synthesis conditions.

To better control the anisotropic growth of Au microparticles and obtain uniform RSMPs as SERS substrates, three parameters including the concentrations of NAAN, PVP, and the reaction time were investigated. As shown in Figure 4A–C, the change of NAAN concentration from 3 to 5 mg/mL does not lead to the obvious change in RSMPs, and the corresponding histogram (Figure 4D) illustrates the diameters of 9.54 ± 1.01, 9.90 ± 1.31, and 9.05 ± 1.54 μm, respectively. As a shape control agent, PVP has a high binding ability to Au (111) facets, and its presence is important for the growth of Au microparticles. In the presence of 0.40 and 3.20 mg/mL PVP, the resulting RSMPs exhibit soft lamellae structures (Figure 4E,F). Further increasing the PVP concentration to 12.8 mg/mL results in the RSMPs containing few Au nanoplates with a decreased diameter to 4 μm (Figure 4G). The histogram of corresponding samples represents the RSMP diameter of 11.50 ± 1.11, 8.65 ± 1.90, and 4.40 ± 0.84 μm, respectively (Figure 4H). This demonstrates that a low concentration of PVP (0.4 mg/mL) is favorable for the formation of RSMPs. When the reaction was conducted for 24, 72, and 120 h (Figure 4I–K), the collected RSMPs showed similar morphology and average size (11.46 ± 1.08, 11.98 ± 0.99, and 12.69 ± 1.74 μm, Figure 4L). However, the Au microplates become thick and fold inwards or merge in a continuous stacking process as the reaction time increases, which can eliminate the nanogaps and is unfavorable for SERS enhancement. Therefore, 24 h was set as the optimal reaction time.

**Figure 5:**
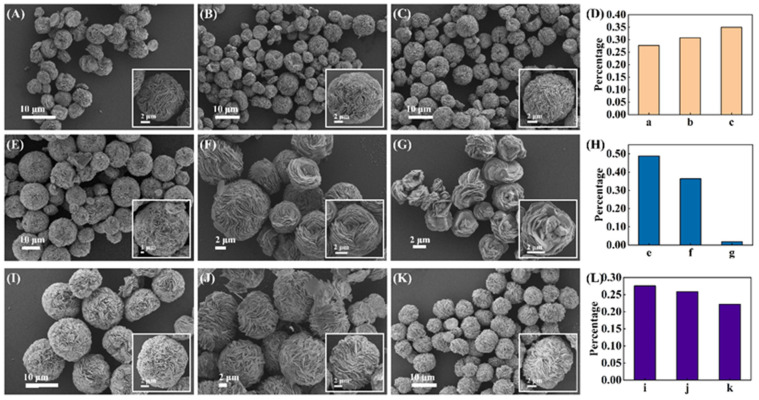
SEM images of RSMP proportion under different conditions. (**A**–**C**) RSMP prepared with 3/4/5 mg/mL NAAN, 1.6 mg/mL PVP, 24 h. (**E**–**G**) RSMP prepared with 0.4/3.2/12.8 mg/mL PVP, 5 mg/mL NAAN, 24 h. (**I**–**K**) The time of RSMPs preparation was 24/72/120 h, respectively, with 0.4 mg/mL PVP, 5 mg/mL NAAN; (**D**,**H**,**L**) Histogram of the proportion of the generated particles under different synthesis conditions.

In addition, we investigated the yield of the resulting RSMPs prepared at different reaction conditions. As shown in Figure 5A–C, the proportion of satisfactory particles slightly increased with the increase of NAAN concentration, and the corresponding histogram in Figure 5D shows the percentages of 27.66%, 30.75%, and 34.92%. As the PVP concentration increases from 0.4–3.2 mg/mL, the percentage of RSMPs decreases from 48.78% to 36.36% (Figure 5E). Further increasing PVP concentration up to 12.8 mg/mL results in a mixture of small gold-based microparticles (Figure 5G), which is caused by the fast reduction of HAuCl_4_ to Au atoms in the presence of excessive PVP. Therefore, the presence of PVP for the formation of RSMPs is necessary, while an overly high concentration is unfavorable. As shown in Figure 5I–K, the corresponding yields are 27.59%, 25.89%, and 22.15% for the reaction times of 24, 72, and 120 h, respectively (Figure 5L), revealing that the reaction time has less effect on the yield of RSMPs. Based on the results mentioned above, we can conclude that the effects of NAAN concentration and synthesis time on RSMP morphology are not significant, while the concentration of PVP has a dramatic impact on the particle size and morphology. In order to obtain SERS substrates with uniform size and morphology, we finally chose 5 mg/mL NAAN, 0.4 mg/mL PVP, and a synthesis time of 24 h as the optimal condition for the preparation of RSMPs.

**Figure 6:**
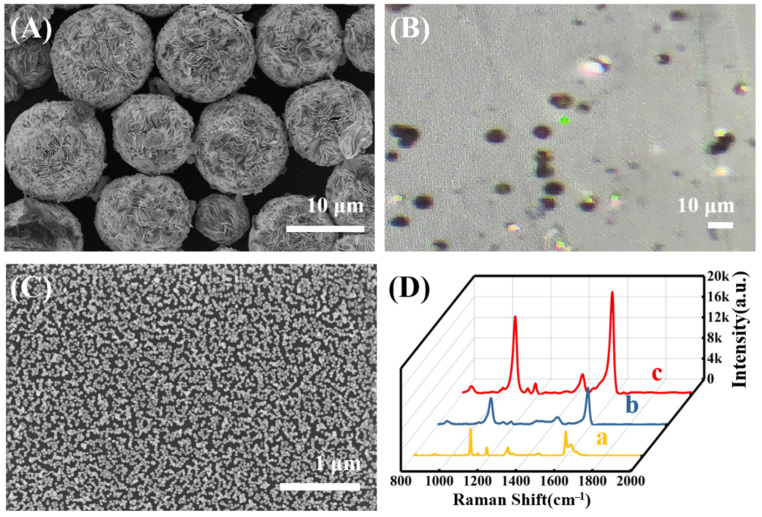
The characterization of RSMP, (**A**) SEM, (**B**) optical microscope. (**C**) SEM image of commercial Raman chip. (**D**) The Raman spectrums of 4-MBA powder (line a), commercial chip@4-MBA (line b), and RSMP@4-MBA (line c).

The microplates and the densely-packed layered structures provide a large number of sharp edges as well as nanogaps among different layers, which can generate hotspots for electromagnetic enhancement (EM) [31]. In order to evaluate the SERS enhancement of RSMPs, commercial SERS chips with SERS enhancement factors (EF) of 1.0 × 10^4^ were applied for the comparison (Xiamen Spectroscopy Scientific Instruments Co., Ltd., Xiamen, China). The average size of RSMPs is 11.5 ± 1.1 μm (Figure 6A), which is much larger than the diameter of the Raman laser spot size (about 2 μm, Figure 6B), allowing the measurement of SERS signals on an individual RSMP. In contrast, the Raman chips were prepared by the self-assembly of spherical Au nanoparticles (NPs) (diameter of 44–56 nm) into a well-organized array (Figure 6C). After the modification of 4-MBA via the Au-S bond, the SERS intensity using RSMPs as SERS substrate is almost four times higher compared to that using the commercial chip as SERS substrate (Figure 6D), revealing that the as-prepared RSMPs demonstrate a better enhancement effect. The corresponding SERS enhancement factors (EF) were calculated to be 2.8×10^6^ for the RSMPs, according to the reported method [24].

Thanks to the superb SERS enhancement, RSMPs can be utilized for ultrasensitive H_2_O_2_ detection. Hydrogen peroxide (H_2_O_2_) is a key redox signaling agent produced under the control of growth factors and cytokines. At low physiological levels in the nanomolar range, H_2_O_2_ is the primary agent signaling through specific protein targets, and it participates in metabolic regulation and stress response to support cell adaptation to changing environments and stress [32]. A large number of studies have focused on the analysis or imaging of intracellular H_2_O_2_ levels [33,34,35,36,37]. In addition, it has been suggested that extracellular H_2_O_2_ is also a fundamental signaling molecule, and it could activate related pathways to cause the corresponding response or the progress of tissues, leading to tumor progression, vascular damage, and even Alzheimer’s disease [38,39,40]. To better understand the production and role of H_2_O_2_ in physiology and disease, it is important to achieve sensitive detection of physiological extracellular/exogenous H_2_O_2_.

Research has been carried out for H_2_O_2_ detection. In particular, phenylboronic acid was mostly applied as a probe since it could selectively react with H_2_O_2_ to produce phenol. Several sensing methods were developed based on this reaction including electrochemical luminescence (ECL) [41,42], fluorescence (FL) [43,44,45], chemiluminescence (CL) [46,47,48,49] and SERS [50,51,52]. Among these methods, SERS has attracted great attention due to its ability to characterize vibrational peaks, insusceptibility to photobleaching, and unmatched detection sensitivity [3]. The typical SERS sensing of H_2_O_2_ is based on the mercaptophenylboronic acid (MPBA) self-assembled monolayer on the SERS substrate. The presence of H_2_O_2_ leads to the transformation of the phenylboronic acid group into the phenol group, resulting in the major shift of typical C=C stretching vibration from 1569 cm^−1^ to 1600 cm^−1^ [53]. However, this SERS sensor has shown some major drawbacks in two ways: (1) the readout signal was recorded based on the shift of the phenyl group, but a slight shift of the benzene group will lead to the overlap of these two peaks, and it is difficult to distinguish this change, resulting in severe error. (2) The SERS signals were collected in the range of 1000–1700 cm^−1^, where most of biomolecules may generate strong interference signals from the lipid and proteins, significantly hindering the SERS measurement. Therefore, the interference could be efficiently eliminated or avoided, which could lead to false-positive results as characteristic peaks located in the “fingerprint” region were used as analysis standard. To overcome these limitations, we further function the phenol group with a new compound 4-DP via the azo coupling reaction, the introduction of alkyne group results in a new Raman shift in 2125 cm^−1^ which is located in the Raman-silent region and the surface ligand of SERS substrate or biomolecules have no Raman signal in this range [54,55,56]. This results in a high signal/noise ratio, and greatly improves the sensitivity of the H_2_O_2_ sensor.

**Figure 7:**
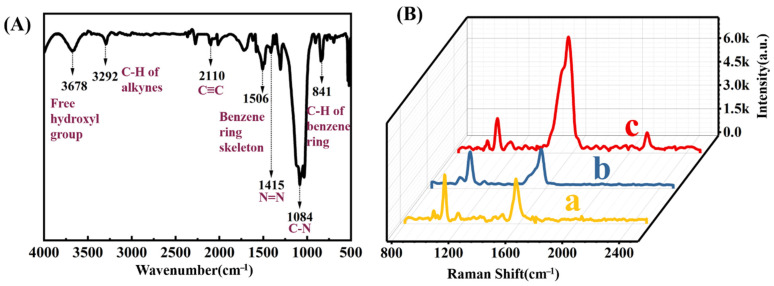
(**A**) FT-IR spectrum of 4-DP; (**B**) the Raman spectrum of RSMP@4-MPBA (line a), probe+4-DP (line b), probe@H_2_O_2_@4-DP (line c).

4-DP was synthesized according to the diazonium reaction, and its IR spectrum displays a characteristic absorption peak of 1415 cm^−1^, which is ascribed to the N=N stretch of the diazonium group (Figure 7A) [57]. The absorption peaks at 1506 cm^−1^, 1084 cm^−1,^ and 2110 cm^−1^ are due to the stretching of benzene, C-N, and C≡N groups, respectively. After the modification of 4-MPBA on RSMPs, two Raman peaks at 1071 and 1569 cm^−1^ were observed, which are attributed to the plane deformation of C-H and the stretching vibration of C=C on the benzene ring (Figure 7B line a). 4-DP could not be attached to the RSMPs or 4-MPBA directly since there was no new Raman peak after the incubation with SERS probes (Figure 7B line b). Upon the reaction with H_2_O_2_, the peaks at 1569 cm^−1^ become slightly broadened with a side peak at 1600 cm^−1^, which is consistent with the reference [53]. Apparently, most of the newly-formed peak overlaps with the original peak, resulting in a difficult separation. After further incubation with 4-DP, new peaks due to the formation of azobenzene located at 1141, 1391 and 1438 cm^−1^ can be observed, revealing the formation of azobenzene (Figure 7B line c). However, the peaks in this range suffered from the substantial interference from the background signal of RSMP, and the accurate measurement of its intensity for the H_2_O_2_ detection is difficult. Fortunately, an additional peak located at 2125 cm^−1^ was observed, which is due to the presence of alkyne group, enabling the high signal/noise determination of H_2_O_2_.

**Figure 8:**
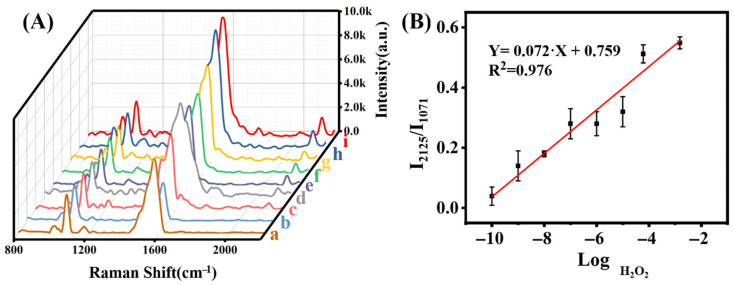
(**A**) The Raman spectrum of RSMP@4-MPBA that reacted with different H_2_O_2_ concentrations of (a–i): 0, 0.1 nM, 1 nM, 10 nM, 100 nM, 1 μM, 10 μM, 60 μM and 1.5 mM, respectively, then connected with 4-DP; (**B**) The calibration curve of I_2125_/I_1071_ ratio of concentration of H_2_O_2_ from 0.1 nM to 1.5 mM.

After incubation of the 4-MPBA with different concentrations of H_2_O_2_ and subsequent reaction with excess 4-DP, their SERS spectra were measured and are shown in Figure 7A. As the H_2_O_2_ concentration gradually increased, the intensity of the SERS peak at 2125 cm^−1^ increased. Therefore, we selected two characteristic peaks at 2125 cm^−1^ and 1071 cm^−1^ and used the ratio of their corresponding intensity for the H_2_O_2_ detection. As shown in Figure 8B, I_2125_/I_1071_ is linearly correlated to H_2_O_2_ concentration in the concentration range of 0.1 nM–1.5 mM (Figure 8A), and the calibration equation is Y = 0.072X + 0.759 with R^2^ of 0.976 (Figure 8B). The corresponding limit of detection (LOD) is 0.1 nM, which is much lower than those reported in the previous work (Table 1). The significant improvement is owing to the high EF of the SERS substrate; more importantly, the introduction of Raman peak in the silent range plays a crucial role.

**Figure 9:**
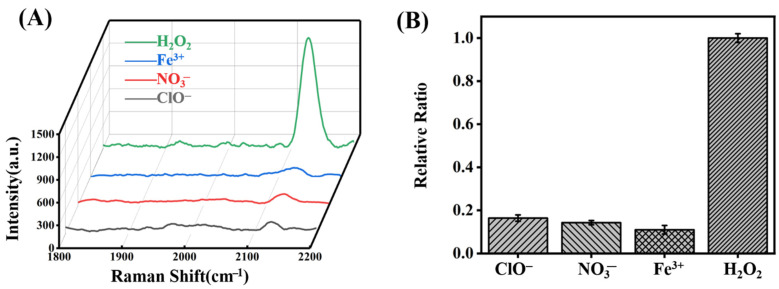
(**A**) Raman spectra of probes reacted with ClO^−^, NO_3_^−^, Fe^3+^ and H_2_O_2_ in the range of 1800–2200 cm^−1^ in the silent region; (**B**) Normalizing the intensity of the peak at 2125 cm^−1^ after reaction with 0.06 mM H_2_O_2_ and the relative ratios of 10 mM of ClO^−^, NO_3_^−^ and Fe^3+^.

Next, we investigated the selectivity of this sensor by testing the response of ClO^−^, NO^3−,^ and Fe^3+^. After incubation of the SERS probe with 10 mM of each sample and subsequent reaction with 4-DP, their corresponding SERS spectra are shown in Figure 9A. It can be seen that these three interferences can only induce slight SERS signals. A comparison with the spectra of the blank SERS probe molecule and the probe molecule after reaction with 0.06 mM H_2_O_2_ in the silent region is shown in Figure 9B. Normalizing the intensity of the peak at 2125 cm^−1^ after the reaction with 0.06 mM H_2_O_2_, the relative intensities of ClO^−^, NO^3−,^ and Fe^3+^ are 0.16, 0.14, and 0.10, respectively (Figure 9B). The concentration of interferents in the experiment is about 166 times that of H_2_O_2_, but the peak intensity at 2125 cm^−1^ was still much less than that of H_2_O_2_, demonstrating that our SERS probe was able to specifically identify H_2_O_2_.

In order to verify the feasibility of this method in a biological environment, the determination of H_2_O_2_ in human serum was conducted via the typical additional method. Simply put, the human serum was diluted 1000-fold by PBS, followed by the addition of H_2_O_2_ with known concentrations. After the treatment of the SERS probe with prepared H_2_O_2_ solution and 4-DP, their corresponding SERS spectra were measured. As shown in Table 2, the recovery rates were in the range of 96.52% and 116.62% with relative standard deviations (RSDs) less than 9.22%. This result reveals that the as-developed SERS sensor could be applied for the clinical determination of H_2_O_2_.

## 4. Conclusions

In this study, we have successfully developed a new SERS method for the specific detection of H_2_O_2_ based on the Raman-silent region signal molecule. The resulting SERS sensor demonstrated excellent performance in terms of high sensitivity, broad detection range, and good selectivity. Owing to the good capability against interference, especially the near-zero signals of biomolecules in the Raman-silent range, we envision that this strategy can be applied for the sensitive and specific monitoring of the various physiological processes that produce H_2_O_2_.

## Figures and Tables

**Table 1:** H_2_O_2_ detection of other SERS methods VS. our method.

Method	Material	Linear Range (M)	Limit of Detection (M)	Reference
Electrochemiluminescence	Hydrogel composite	1.0 × 10^−8^–5.0 × 10^−5^	2.9 × 10^−9^	[42]
Electrochemiluminescence	Modified Electrodes	1.0 × 10^−5^–5.0 × 10^−3^	4.3 × 10^−6^	[58]
Electrochemistry	Hybrid nanoflower	2.0 × 10^−8^–3.6 × 10^−6^	7.0 × 10^−9^	[59]
Electrochemistry	Graphene oxide nanocomposite	5.5 × 10^−7^–5.2 × 10^−4^	8.8 × 10^−9^	[60]
Fluorescence	Arylboronate-pyridinium	0–1.5 × 10^−5^	5.6 × 10^−6^	[43]
Fluorescence	HKPerox-Red	0–1.0 × 10^−4^	4.8 × 10^−9^	[61]
Colorimetry	Oxidized HRP and ABTS	5.0 × 10^−7^–6.5 × 10^−5^	1.7 × 10^−9^	[62]
Colorimetry	Papain and TMB	55.0 × 10^−6^–9.0 × 10^−5^	2.1 × 10^−6^	[63]
SERS	Gold Nanorod	1.0 × 10^−6^–10^−4^	3.0 × 10^−7^	[52]
SERS	AuNPs	1.0 × 10^−7^–2.5 × 10^−6^	7.0 × 10^−8^	[64]
SERS	AuNPs	1.0 × 10^−7^–2.0 × 10^−5^	8.0 × 10^−8^	[53]
SERS	AgNPs	1.0 × 10^−6^–10^−2^	1.0 × 10^−6^	[65]
SERS	ZIF-8	1.0 × 10^−9^–10^−3^	3.6 × 10^−10^	[66,67]
SERS	AuNPs	1.0 × 10^−6^–10^−4^	2.0 × 10^−7^	[66]
SERS	RSMPs	1.0 × 10^−10^–1.5 × 10^−3^	1.0 × 10^−10^	this work

**Table 2:** SERS detection of H_2_O_2_ in serum (*n* = 3).

Sample	Spiking Conc. (mM)	Detected Conc. (mM)	Recovery (%)	RSD (%)
1	1.200	1.180 ± 0.240	98.640	6.630
2	0.500	0.580 ± 0.120	116.620	9.220
3	0.010	0.009 ± 0.003	96.520	1.760

## Data Availability

Not applicable.
